# Overcoming obstacles to implementing a primary care research framework

**DOI:** 10.1186/1750-4732-1-4

**Published:** 2007-02-01

**Authors:** Roberto Cardarelli, Margaret Seater, Elizabeth Palmarozzi

**Affiliations:** 1 Department of Family Medicine, Division of Education and Research, Texas College of Osteopathic Medicine, University of North Texas Health Science Center at Fort Worth, Fort Worth, Texas, USA

## Abstract

**Background:**

Primary care research has recently garnered greater attention at the national level. Yet, primary care (i.e., family medicine, internal medicine, pediatrics, and obstetrics and gynecology) departments within academic institutions struggle to develop and sustain strong research frameworks.

**Methods:**

This paper discusses a successful model that was developed in the department of family medicine at the University of North Texas Health Science Center at Fort Worth/Texas College of Osteopathic Medicine.

**Results:**

Overall, the framework revolves around three core values: training future primary care researchers, providing resources to emerging and junior faculty members, and creating a partnership with the community and clinicians to conduct primary care clinical research.

**Conclusion:**

Significant effort is required to establish a successful research framework in family medicine. The framework presented herein serves as an example for other departments to use and adapt in developing their research division.

## Background

Primary care is a major entry point to the health care system, therefore the decisions primary care clinicians make determine if health care resources are used appropriately [1]. Family medicine clinicians, in particular, have some of the highest numbers of patient visits per year in the country [2]. Historically, family medicine has not been considered a significant contributor to research. Today, family medicine and other primary care specialties have garnered the attention of academic and federally-funded researchers by developing practice-based research networks (PBRNs). The Future of Family Medicine report stresses that the profession must make its own niche, including conducting research that impacts family medicine and the communities the clinicians serve [3]. In this paper, we share the successes and challenges of developing a research framework in the department of family medicine at the University of North Texas Health Science Center at Fort Worth (UNTHSC), Texas College of Osteopathic Medicine (TCOM).

## Methods

The Division of Education and Research (DEAR) in the department of family medicine at UNTHSC was established in 1997 with a vision to foster an environment conducive to conducting primary care research. The following goals and objectives were established:

### Goal 1. Offer clinicians educational opportunities to better understand the research process and how to improve the provision of care

Objective 1.1. Design and implement an evidence-based medicine (EBM) curriculum.

Objective 1.2. Offer continuing medical education credit for all EBM lectures.

Objective 1.3. Create a website with PowerPoint^® ^lectures and links to EBM resources.

Objective 1.4. Ensure EBM educational opportunities are offered regularly.

### Goal 2. Create a "population laboratory" to perform research that is pertinent to family medicine/primary care and the surrounding communities

Objective 2.1. Create a practice-based research network in North Texas.

Objective 2.2. Establish scientific review and community advisory boards to ensure the research agenda is pertinent to primary care and the communities.

Objective 2.3. Foster interdisciplinary support within the institution, including administration.

Objective 2.4 Conduct research to better understand and eliminate health and health care disparities.

### Goal 3. Train medical students to become primary care researchers

Objective 3.1. Secure funding to implement a primary care clinical research program.

Objective 3.2. Recruit medical students into the program as they enter medical school.

Objective 3.3. Ensure students obtain the necessary skills to conduct high-quality research and have access to mentors.

DEAR formed a three-prong research framework to achieve these objectives: (1) the Center for Evidence-Based Medicine (CEBM), (2) the North Texas Primary Care Practice-Based Research Network (NorTex), and (3) the Primary Care Clinical Research program (also referred to as the pre-doctoral clinical research fellowship). The CEBM is both an educational forum and research program for students, residents, and practicing clinicians. NorTex is a practice-based research network that collaborates with university members, local clinicians, patients, and surrounding communities to conduct participatory research. The primary care clinical research program trains medical students to become primary care researchers (they receive a Master of Science in Clinical Research).

## Results

### Center for Evidence Based Medicine

The Center for Evidence-Based Medicine (CEBM) was established to teach and conduct research related to EBM, which is a formal method of translating research into practice [4]. It uses the best current evidence to assist in making clinical decisions. EBM also takes into account the patient's wishes, values, and expectations. The CEBM is headed by a director and assisted by a research associate and administrative assistant. The CEBM was established in 2004 and is currently responsible for an EBM faculty development lecture series, teaching medical students and residents, and conducting EBM related research projects and scholarly activities.

To meet these aims, an EBM curriculum was created to provide instruction on how to ask answerable questions, search the medical literature, choose relevant studies, and assess the evidence for its validity, importance, and applicability (Objective 1.1). The EBM curriculum is taught in a journal club format rather than didactic lectures alone. The curriculum is composed of 6 sessions (each session is approximately 1 hour and 30 minutes) as shown in Table 1. The first 45 minutes of the session is a PowerPoint^® ^presentation and the remainder of the session is used to appraise an article and discuss its applicability to clinical care. This allows participants to immediately apply the principles that were learned. They are also provided another article to critique and read before the next class. Worksheets are used to structure the discussions and train participants to be systematic in their evaluation and assessment of the literature. Examples of the lectures and worksheets can be found at the CEBM's website (Objective 1.3) [5]. Evaluations of the lectures found that participants appreciated the small classroom environment. Additionally, this EBM course gives participants exposure to landmark studies that shape current controversies in diagnosis and treatment. Clinicians receive one category 1 American Medical Association or 1A American Osteopathic Association CME credit for each lecture (Objective 1.2). These sessions have been offered in the 2004–2005 and 2005–2006 academic years (Objective 1.4) and will be offered yearly.

**Table 1:** Evidence-Based Medicine Curriculum

Lecture Topic	Hours
Introduction to EBM	1.5
Basic statistics	1.5
Critical appraisal of articles on diagnosis	1.5*
Critical appraisal of articles on prognosis	1.5*
Critical appraisal of articles on therapy	1.5*
Applying the evidence	1.5*

* These sessions are comprised of a 45-minute didactic lecture followed by a 45-minute journal club workshop

Faculty members are encouraged to use EBM principles with students during clinic and daily hospital rounds. Faculty are encouraged to move away from asking ambiguous factoids (i.e. "pimp" questions), such as "What is the Cushing's reflex?," instead asking students such questions as the sensitivity and specificity of imaging modalities, the absolute risk reduction of tPA in thrombotic stroke, and the applicability of evidence to a particular patient. These are sophisticated questions that require investigation rather than recall. In this context, EBM is being used to move students beyond obtaining the correct diagnosis to formulating and following through with a treatment plan. The CEBM does not dictate the exact format for teaching EBM to students and residents, but strongly encourages using the principles that are taught in the EBM curriculum to ensure a continuity of learning (Objective 1.4). The CEBM offers the training and resources for the faculty to teach EBM, including how to search and locate evidence on the Internet. EBM prescription pads [6] are provided to students and residents to help guide them in creating EBM-specific questions. Faculty members are encouraged to review and use them daily as teaching points and apply them to the patients.

The CEBM has also published papers on how to teach EBM to medical students during family medicine rotations [7] and has conducted research on the accuracy of pharmaceutical advertising [8]. To secure funding and to conduct interventional studies related to EBM has been a great challenge. Time, personnel, research faculty, and funding opportunities would aid in overcoming these obstacles.

### The North Texas Primary Care Practice-Based Research Network (NorTex)

NorTex, a practice-based research network (Objective 2.1), was formed to function as a laboratory to serve the common goal of clinicians, researchers, and community members – to improve the health of their community. This endeavor began by seeking support from the institution, including its president, vice-president, deans, and other leaders. The importance of creating NorTex was recognized and seed-funding was provided to assist in NorTex's recruitment efforts (Objective 2.3). Recruitment efforts consisted primarily of mail-outs to all primary care clinicians (family medicine, general internal medicine, pediatrics, and obstetrics and gynecology) in several counties in north Texas. These mail-outs included information about NorTex and membership forms. A NorTex website was also developed to help disseminate and post information [9].

NorTex's organization includes of a director, research coordinator, and the scientific review and community advisory boards (Objective 2.2). These boards' primary function are to maintain the integrity of the research agenda and to approve or reject proposals that are submitted to NorTex. This ensures a balanced research agenda and validates the importance, scientific merit, and feasibility of the projects. The scientific review board (SRB) is comprised of experts in the areas of research design, methodology, and biostatistics from the medical school, school of public health, and graduate school of biomedical sciences. The community advisory board (CAB) consists of community leaders and representatives of NorTex member organizations, such as the county hospital district and health department.

Currently, NorTex has over 54 member clinics and 105 individual members from the medical school, school of public health, graduate school of biomedical sciences, county health department, private clinics, and community leaders. Members meet regularly, either in-person or by conference call. Current studies include understanding how life stressors are related to cardiovascular disease (National Institutes of Health funded study), the impact of psychosocial factors on highly-active antiretroviral therapy (HAART) among individuals infected with HIV (UNTHSC intramural study), and ethnic differences in visceral abdominal fat as risk factors for cardiovascular disease (National Institutes of Health funded study) (Objective 2.4).

### Primary Care Clinical Research Program

Sustaining primary care research relies on training medical students in an environment that values research and provides career tracks for investigators. The primary care clinical research program offers students a Master of Science in Clinical Research which is completed during the four year Doctor of Osteopathy curriculum. This program began in 2003 and was funded by a pre-doctoral training grant from the Health Resources and Services Administration (Objective 3.1). It is now in the process of being institutionalized at UNTHSC. The program selects about two incoming medical students per year. The recruitment strategy consists of mail outs, a website [10], word-of-mouth, and a presentation during student orientation week. Applicants are required to submit an essay and provide transcripts and Medical College Admission Test scores. These applicants are thoroughly screened by the program faculty and only highly qualified applicants are invited for an interview. Currently, there are 8 students in the program. The program celebrated its first graduate in May 2006 (Objective 3.2). The grant partially covers the salaries of a research associate, administrative assistant, fellowship director, and several faculty members involved with advising and teaching courses. Each student receives a stipend to help offset the cost of graduate school courses and a small monetary award to help cover research costs (copies, supplies, etc.). Each student is assigned to an advisor/mentor and is required to have an approved degree plan early in their first year.

The curriculum includes coursework in biostatistics, epidemiology, research methods, scientific communication, evidence-based medicine, and ethical and legal issues in research (Table 2), as well as two small-scale projects and an intensive thesis (Objective 3.3). Students take these courses primarily in the evening and during the summer when medical school is not in session. Students are mentored throughout the research process, which includes formulating a study question, conducting a literature review, building a protocol, applying for funding, submitting an institutional review board application, collecting and analyzing data, and writing a manuscript for publication. The curriculum is designed to develop critical thinking and cultivate a positive research attitude among clinician-researchers in training. Mentoring is highly emphasized to facilitate the vigorous four-year curriculum these students must endure.

**Table 2:** Primary Care Clinical Research Curriculum*

Course	Credit Hours
Biostatistics I	3
Biostatistics II	3
Scientific Communications	3
Introduction to Clinical Research & Studies	3
Ethical, Legal and Social Issues for Responsible Clinical Research	1
Principles of Epidemiology	3
Family Medicine Research Colloquium	3
Special Problems in Family Medicine Research	6
Thesis	6
**Total**	**31**

* Advanced credit hours from medical school: 6

Students study topics of their choosing and thesis committees are established to provide guidance. Students have studied important primary care questions such as:

• Does osteopathic manipulative treatment relieve low back pain? [11]

• Does the patient-doctor relationship account for health disparities?

• Does acculturation affect glycemic control among Mexican-Americans with Type II Diabetes?

• Do experiences of racism impact preventive screening utilization?

The recasting of family medicine as a specialty with research capabilities promises to increase medical students' interest in family medicine as a career.

## Discussion

DEAR has been successful in each of its three initiatives; the CEBM, NorTex, and the primary care clinical research program. As noted above, DEAR has been fortunate in securing funding, publishing articles, and training students and clinicians to be clinical researchers. However, challenges had to be overcome in creating a research framework in family medicine.

Explaining why family medicine physicians have avoided research until recently, John Howie said, "Those who deliver health services work in a culture that values and rewards doing rather than conceptualizing. Many have become general practitioners because they have rejected the apparently research-centered values of teaching hospitals and their staff" [12]. On the other hand, many in the primary care community have begun to recognize that family medicine is the cornerstone of the health care system and there can be little overall improvement in the system without improving family medicine [13]. The health of a nation, states, and cities has been shown to be a reflection of the primary care infrastructure of the respective areas [14]. As the first point of contact for patients seeking health services, family medicine physicians are in a position to study factors that impact health, such as the cost of healthcare, common and serious diseases, the decision-making processes of patients, and the impact of health and illness on patients and providers [12,13]. As Murray Tilard said, "We do research because we need practical answers to practical questions" [12].

In creating the CEBM, clinicians, residents, and students individually struggled to determine how they could use EBM in their clinical practice. For students, clinical inexperience limits their ability to identify how study populations may differ from the patients they see in clinic. Students find it difficult to judge the generalizability of studies because of their limited clinical experience. Therefore, it is important and critical to ensure learning EBM is a process throughout the four-year curriculum. Repetition is key to internalizing EBM concepts. In addition to the EBM curriculum described earlier, faculty must apply the principles with students and residents in the clinic and hospital setting as they care for patients. Because medical schools do not teach statistics in great detail [15], special attention is needed to help interpret data appropriately. We have designed a "basic statistics" lecture as part of the EBM curriculum. For practicing clinicians, old habits die hard. By offering the EBM curriculum yearly, faculty will be exposed to EBM concepts multiple times. As an electronic health record system is incorporated, clinicians may be more inclined to adopt EBM because of greater access to the Internet.

In developing NorTex, balancing the sometimes competing goals of research and patient care was a challenge. These were addressed by minimizing the burden on the clinicians and their staff. Research assistants, rather than clinic personnel, recruit participants, conduct all informed consents, and control all aspects of the NorTex research studies. Participating clinics choose their level of involvement within a study. For example, some clinics may place flyers in their waiting rooms while other clinics volunteer a staff member to recruit study participants. In addition, grants may cover the time and effort of clinic staff and the clinician who are involved in the research project. Mold et al have argued that in order to resolve the tension between clinical care and conducting research studies, PBRNs should evolve from "laboratories" into "collaborative learning environments" [16]. By advancing this view, the aim is to bridge traditional distinctions between research and quality improvement. They contrast the values of research and quality improvement by pointing out that the intent of academic research is discovery, while the intent of quality improvement is application.

Mold et al are correct to call attention to the fact that medical progress is slow [16]. Balas and Boren estimated that it takes 17 years to turn 14% of original research into significant changes that benefit the patient [17]. It has been suggested that disseminating discoveries through workshops and list-serves may be more efficient than sharing information through traditional forms of communication such as journals [16]. These inefficiencies in medical research certainly warrant closer examination. However, there are many merits to the academician's approach. The process of peer review, the tools of statistics, and the principles of sound study design help the profession separate truth from perception.

These competing visions of PBRNs as academic laboratories and quality improvement departments underscore the need for collaboration. From the university's perspective, staying closely connected to the community makes research more relevant. From the clinician's perspective, university faculty members provide valuable technical expertise. NorTex has found collaboration to be a mutually enlightening experience. The key has been preventing either party from dominating the research agenda through its strategy of establishing the scientific review and community advisory boards. These boards assess the scientific merits of the proposed projects while ensuring the project's importance to primary care and the community.

The primary care clinical research fellowship has focused its efforts in minimizing attrition, improving the curriculum, and ensuring students complete the program within four years. Recruiting and retaining students has been an important problem to address. Each year, a variable number of students have enrolled in the program. The first student graduated in May 2006, with the second student expected to graduate in December 2006. There are eight students currently in the program. Three students left the program, while one student started the program a year late and is willing to take the extra year to complete the program. Retention has improved by providing necessary guidance and resources to the students, such as assigned advisors, a research project timeline, quarterly meetings, encouragement to present their research findings, and a student conference room. Staff were given adequate time to manage required paperwork, schedule regular meetings between students and their mentors, and oversee students' required four-year plans. The research conference room is used as both as a classroom and as a place to work on projects. This conference room is furnished with audiovisual equipment, computers, printers, statistical and reference management software, a library full of research reference books, file cabinets, video conferencing capabilities, and telephones. This conference room has symbolic importance in that it is a designated physical space where students devote time to earning their degree. To continue the program's retention efforts for the next academic year, an induction ceremony is planned with a speaker to bring the message to students that research is important for the future of medicine.

With respect to scouting prospective clinical research students, brochures were sent to the incoming medical school class. In the future, the plan is to screen medical school applicants for prior research experience and begin the recruitment process during the interview season. Recruiting earlier might improve participation. Stipends have been offered to offset the cost of tuition to help improve participation. Fellows are encouraged to participate in an elective research rotation during their third or fourth year of medical school. This provides protected time for them to work on their research projects. Recruitment and retention issues underscore the importance of institutionalizing the fellowship.

The program entrusts more authority to the students than is usually encountered in academia. As noted, students are the captains of their own projects from beginning to end. However, some may question the quality of student projects. Training students is an investment for the future that will pay dividends by increasing students' interests in primary care, research, and, ultimately, better patient care. It is anticipated that the fellows will make better residents and clinicians, capable of thinking critically and making a meaningful contribution to the medical profession.

## Conclusion

In establishing a primary care research framework at our institution, obstacles had to be overcome to succeed. The Center for Evidence Based Medicine has proven to be mutually beneficial to teachers and learners by revitalizing our professional commitment to perpetual learning. Future goals are to demonstrate its benefit to patients through research. The practice-based research network, NorTex, has started a two-way exchange of ideas between physicians and communities that will transform both traditions. Yet, stronger ties are needed with the communities and to ensure that the research conducted is a reflection of their needs. Finally, the primary care clinical research fellowship program has graduated its first student and it is currently training eight other students who will sustain the family medicine research culture in the future.

Institutions have their own strengths and weaknesses. The intention of the proposed framework is not for it to be duplicated, but rather to be molded to fit a department's environment, infrastructure, and needs. Our primary recommendation to departments of family medicine is to acquire support from all members in the department, including the chair and dean(s) of the medical school. The "return-on-investment" can be measured through published manuscripts, state and national conference presentations, and federal and non-federal funding. These achievements result in institutional recognition at the local, state, and national levels. It is imperative that resources are allocated to establishing a research division, including space, faculty time, and a designated administrative assistant and research associate. With these basic resources and support the research framework has the opportunity for growth.

## Abbreviations

CEBM: Center for Evidence-Based Medicine

CME: Continuing medical education

DEAR: Division of Education and Research

EBM: Evidence-based medicine

HAART: Highly-active antiretroviral therapy

HIV: Human immunodeficiency virus

NorTex: North Texas Primary Care Practice-Based Research Network

PBRN: Practice-Based Research Network

TCOM: Texas College of Osteopathic Medicine

UNTHSC: University of North Texas Health Science Center at Fort Worth

## Competing interests

The author(s) declare that they have no competing interests.

## Authors' contributions

RC is the director for the Division of Education and Research, Center for Evidence-Based Medicine, and the North Texas Primary Care Practice-Based Research Network. RC drafted the manuscript.

MS drafted the manuscript and is actively conducting research in the Division of Education and Research and is a student in the Primary Care Clinical Research Program.

EP is the chair of the Department of Family Medicine. EP drafted the manuscript.
